# Root-zone improvements in spinach through commercial biostimulants application vary among cultivars

**DOI:** 10.3389/fpls.2026.1830913

**Published:** 2026-07-13

**Authors:** Md Noor E Azam Khan, Fahmida Fiza, Joseph Masabni, Genhua Niu

**Affiliations:** Texas A&M AgriLife Research, Dallas, TX, United States

**Keywords:** greenhouse production, humic acid, nutrient regulation, root-zone management, seaweed extract, *Spinacia oleracea*

## Abstract

Improvements in root-zone conditions can potentially enhance shoot growth and are especially important in controlled environment systems, where maximizing yield within limited production space is essential. Commercial seaweed extract (SW) and humic acid-based biostimulants (HA) are widely used to improve root function and nutrient uptake; however, information regarding their effects through root-zone application in spinach (*Spinacia oleracea* L.), particularly commercial humic acid-based formulations containing microbial-derived extracts, remains limited. This study evaluated the effects of root-zone application of the commercial SW and HA products (Kelpak^®^ and Nutra Need™, respectively) on growth, phytochemical attributes, and mineral accumulation of three spinach cultivars (‘Lakeside’, ‘Mandolin’, and ‘SV2157’) grown in pots under greenhouse conditions. Treatments included a control (no biostimulant), 1% SW (3 mL·L^-1^), 1% HA (3 mL·L^-1^), and a combined SW+HA treatment (1.5 + 1.5 mL·L^-1^) applied through sub-irrigation. Significant interactive effects between biostimulant and cultivar were observed for shoot and root biomass and mineral content. In ‘SV2157’, all biostimulant treatments increased shoot dry weight by approximately 34% compared with the control, while SW and SW+HA increased root dry weight by approximately 47% relative to the control. These responses were associated with greater mineral accumulation, with HA increasing N, K, Fe, S, and B and SW increasing Ca, Mg, Fe, Mn, and S compared with the control. In contrast, in ‘Mandolin’, HA and SW+HA increased root dry weight by 177–268% compared with the control; however, shoot dry weight increased only under SW, while HA and SW+HA resulted in lower N and Mg accumulation relative to the control. ‘Lakeside’ exhibited no significant root growth responses, and Ca, Fe, and Mn concentrations remained consistently lower across biostimulant treatments compared with ‘SV2157’ and ‘Mandolin’. The combined SW+HA treatment promoted balanced multi-element accumulation but did not consistently result in greater biomass than individual SW and HA applications. Leaf morphological traits were primarily cultivar dependent, whereas secondary metabolites and antioxidant activity showed limited responses to biostimulant treatments. These results indicate that the effectiveness of applying biostimulants in the root zone of spinach is strongly cultivar dependent.

## 1 Introduction

Spinach (*Spinacia oleracea* L.) is a nutrient-dense leafy vegetable rich in vitamins (A, C, and K) and essential minerals (calcium and iron), making it an important component of a healthy diet (Patel et al., 2017; Pasumarthy and Poojitha, 2025). Due to its nutritional value and strong consumer demand, spinach is one of the most popular leafy greens in the United States. Over the last decade (2016-2024), per capita consumption increased by approximately 133%, primarily driven by fresh market sales (Agricultural Marketing Resource Center, 2024; USDA, 2024). However, in recent years (2023 and 2024), the fresh spinach production declined by around 35% resulting in higher market prices (USDA, 2025). This gap between rising consumption and declining production highlights vulnerabilities in the U.S. spinach production system. This production decline is closely associated with the strong geographic concentration of spinach cultivation. More than 90% of U.S. spinach production occurs in California and Arizona under open-field conditions (USDA, 2025). These regions are increasingly affected by prolonged drought, water scarcity, and extreme weather, which reduce yield stability and disrupt seasonal production cycles. Controlled environment agriculture (CEA) has emerged as a promising approach to supplement national spinach production and improve supply stability. However, because CEA systems operate within spatially limited environments under intensive management, maximizing productivity per unit area becomes essential. Achieving this goal requires not only optimization of aboveground factors such as light and temperature, but also careful management of the belowground environment. Improved root development can enhance water and nutrient uptake, which subsequently promotes shoot growth and biomass accumulation (Lynch, 2019; Rogers and Benfey, 2015). Therefore, improving root-zone performance is closely correlated with enhanced shoot productivity (Khan et al., 2025a) and represents a critical factor in maximizing yield potential in CEA systems.

Biostimulants have gained attention as tools to improve plant growth through root-mediated processes. They are defined as “*substances or microorganisms that enhance nutrient use efficiency, root development, and physiological activity rather than directly supplying nutrients”* (du Jardin, 2015). Among commonly used biostimulants, seaweed extracts and humic substances are widely studied because of their positive effects on plant performance. Commercial seaweed extract products are commonly derived from brown seaweed species such as *Ecklonia maxima*, *Ascophyllum nodosum*, *Sargassum* spp., *Laminaria* spp., etc. often contain diverse bioactive compounds, including polyphenols, polysaccharides, alginates, polyamines, amino acids, betaines, vitamins, and phytohormone-like compounds (Khan et al., 2009; Deolu-Ajayi et al., 2022; Patel and Mukherjee, 2021). In research, seaweed extract applications improved root length and dry weight in tomato, indicating its role in promoting root development and overall plant growth (Hussain et al., 2021). On the other hand, humic acid is a complex, heterogeneous mixture of naturally occurring organic macromolecules, including phenolic compounds, lignin-derived fragments, fatty acids, polysaccharide residues, peptides, and quinone-containing structures (Capasso et al., 2007). Humic substances originate from the decomposition of plant, animal, and microbial residues and are shaped by interactions among organic matter, microorganisms, and plant roots (du Jardin, 2015). They enhance root development and nutrient uptake by stimulating plasma membrane H^+^-ATPase activity, improving nutrient availability, and influencing rhizosphere processes (Jindo et al., 2012; Canellas et al., 2015). Accordingly, some commercial humic acid-based products incorporate microbial-derived compounds or culture extracts to mimic these natural rhizosphere interactions and potentially broaden their biostimulatory effects.

In spinach, foliar application of seaweed extract has been reported to enhance plant growth by increasing biomass and leaf number while improving nutritional quality through higher concentrations of essential minerals such as nitrogen (N), phosphorus (P), potassium (K), and magnesium (Mg), as well as bioactive compounds (La Bella et al., 2021; Rouphael et al., 2018). Additionally, seaweed extract is associated with increased phenolic compounds, flavonoids, and antioxidant activity in several vegetables, including spinach, cabbage, and broccoli (Fan et al., 2011; Lola-Luz et al., 2013, 2014). Foliar application of humic acid has been reported to increase plant height, leaf area, and biomass in crops such as bok choy and red leaf lettuce (Anandakumar et al., 2024). In addition to improving nutrient acquisition, humic substances may influence primary and secondary metabolism by modifying carbon allocation and overall physiological activity (Nardi et al., 2002).

Although foliar application of these biostimulants has shown benefits in spinach and other crops, it often results in uneven distribution and primarily stimulates aboveground growth (Durrani et al., 2010; Tahapary et al., 2020; Turan et al., 2022). In contrast, applying biostimulants such as seaweed extract and humic acid directly to the root zone may provide a more stable supply to the roots, promote root development, and improve nutrient uptake. Nevertheless, some studies reported that root-zone applications of both seaweed extract and humic acid in spinach increased leaf number, biomass, and pigment contents (chlorophylls and carotenoids) (Naseri et al., 2021; Azzam et al., 2026). However, it remains unclear how seaweed extract and humic acid-based biostimulants differentially influence root-mediated nutrient acquisition and growth responses among spinach cultivars, and whether their combined application provides additional benefits. Furthermore, because previous studies have primarily focused on humic substances alone or in combination with living microbial inoculants, the investigation on contribution of non-living microbial-derived compounds in commercial humic acid-based formulations remains poorly understood (Calvo et al., 2014; du Jardin, 2015; Yakhin et al., 2017; Savarese et al., 2022; Schoebitz et al., 2016). In addition, the interrelationship between biostimulant-induced changes in nutrient transport to aboveground tissues and subsequent growth responses remains insufficiently investigated. Therefore, we hypothesized that root-zone application of commercial seaweed extract, a humic acid-based biostimulant containing extracts from non-living bacterial, fungal, and yeast cultures, and their combination would enhance root growth, nutrient uptake, mineral accumulation, and subsequent shoot growth in spinach, with the magnitude of these responses depending on cultivar.

Our objective was to evaluate the effects of root-zone application of commercial seaweed extract, the humic acid-based biostimulant, and their combination on growth, morphology, mineral accumulation, and phytochemical attributes of multiple spinach cultivars grown under controlled environment conditions. A further objective was to determine whether cultivar-specific root responses and mineral accumulation are associated with subsequent shoot growth responses.

## 2 Materials and methods

### 2.1 Plant materials and propagation

The study used three spinach cultivars (‘Lakeside’, ‘Mandolin’, and ‘SV2157’) obtained from Osborne Quality Seeds (Mount Vernon, WA, USA) and was conducted in two independent cycles under identical propagation conditions. These cultivars were selected based on our recent study (Khan et al., 2026), in which they were classified as potentially heat-resilient based on their ability to maintain canopy expansion under brief exposure to high temperature. Among them, ‘SV2157’ is characterized by dark, glossy leaves and good yield potential.

Seeds were soaked in 3% hydrogen peroxide for 4 h before sowing to soften and disinfect the seed coat and promote uniform germination. This protocol was adopted based on our previous study (Khan et al., 2026), in which poor germination was observed, and the soaking duration was subsequently optimized through preliminary germination trials (unpublished data). After soaking, two seeds were sown per cell (3.8 cm length × 3.8 cm width × 5.7 cm depth) in 72-cell nursery trays (54 cm length × 27.99 cm width × 7.01 cm depth) filled with BM2 germination medium (Berger, Saint-Modeste, QC, Canada). Product information provided by Berger indicates that the medium consisted of premium selected fine-grade peat moss, fine-grade perlite, fine-grade vermiculite, dolomitic and calcitic limestone, a non-ionic wetting agent, and a standard seedling fertilizer starter charge, with an adjusted pH of 5.2 to 6.0. Trays were maintained in darkness for 3 d at 19.03 ± 1.01 °C [mean ± standard error (SE), n = 42] and covered with humidity domes in a controlled propagation rack (1.22 m length × 0.6 m width × 0.62 m height). Domes were removed once more than 50% of seeds had germinated. Seedlings were then grown under warm white light-emitting diode (LED) fixtures (Arize^®^ R Lynk LED luminaire; Current Lighting Solutions, LLC, Cleveland, OH, USA), providing a photosynthetic photon flux density (PPFD) of 202.51 ± 10.6 µmol·m^-2^·s^-1^ at canopy level (mean ± SE, n = 27) under a 12 h photoperiod. The vapor pressure deficit (VPD) during propagation averaged 0.91 ± 0.20 kPa (mean ± SE, n = 42). Notably, the temperature and VPD values represent combined measurements from both propagation cycles. Seedlings were thinned to one plant per cell 7 d after germination in both cycles. Air temperature and relative humidity were recorded using a CR1000 datalogger (Campbell Scientific, Inc., Logan, UT, USA), and light intensity was measured using a PS-100 spectroradiometer (StellarNet Inc., Tampa, FL, USA).

After 21 d, seedlings were transplanted into square pots (10.2 cm length × 10.2 cm width × 7.6 cm height) containing BM6 all-purpose medium (Berger, Saint-Modeste, QC, Canada). According to the manufacturer, this substrate was composed primarily of premium selected coarse-grade peat moss and coarse-grade perlite and included dolomitic and calcitic limestone, a non-ionic wetting agent, and a starter fertilizer charge, with an adjusted pH of 5.2 to 6.0. Plants were maintained under the same environmental conditions (light intensity, temperature, and VPD) for 2 d to allow acclimatization and recovery from transplanting shock. Throughout the propagation and acclimatization phase, plants were subirrigated with a nutrient solution prepared using a water-soluble fertilizer (20-20–20 general purpose; JR Peters Inc., Allentown, PA, USA). The solution was prepared by dissolving 13 g of fertilizer in 15.1 L of tap water (EC = 0.45 dS·m^-1^; pH = 6.68). Based on the manufacturer’s guaranteed analysis (20% total N, 20% P_2_O_5_, and 20% K_2_O), the calculated nutrient concentrations were approximately 172 mg·L^-1^ total nitrogen (N), including 35 mg·L^-1^ ammoniacal N, 47 mg·L^-1^ nitrate N, and 89 mg·L^-1^ urea N, along with 75 mg·L^-1^ phosphorus (P) and 143 mg·L^-1^ potassium (K). The solution also supplied approximately 0.43 mg·L^-1^ magnesium (Mg), 0.43 mg·L^-1^ iron (Fe), 0.43 mg·L^-1^ copper (Cu), 0.21 mg·L^-1^ manganese (Mn), 0.21 mg·L^-1^ zinc (Zn), 0.11 mg·L^-1^ boron (B), and 0.04 mg·L^-1^ molybdenum (Mo). Nutrient concentrations were calculated from the guaranteed analysis and were not analytically verified. The nutrient solution had an average electrical conductivity (EC) of 1.18 ± 0.08 dS·m^-1^ (mean ± SE, n = 6) and a pH of 6.37 ± 0.05 (mean ± SE, n = 6). The EC and pH throughout the entire experiment were measured using a LAQUAtwin EC-11 conductivity meter and a LAQUAtwin pH-11 compact pH meter (Horiba Scientific, Kyoto, Japan), respectively. During propagation and acclimatization, seedling trays and transplanted pots were repositioned regularly to minimize positional effects and maintain uniform environmental conditions.

### 2.2 Experimental design and treatments

The experiment was conducted from October to December 2025 in a glass greenhouse at the Texas A&M AgriLife Research and Extension Center, Dallas, TX, USA (32.77°N, 96.80°W). After acclimatization, uniform seedlings with at least two true leaves were selected and transferred to the greenhouse for treatment application. The study was arranged as a randomized complete block design (RCBD) consisting of three spinach cultivars [‘Lakeside’ (LS), ‘Mandolin’ (MD), and ‘SV2157’ (SV)] and four biostimulant treatments: control (no biostimulant), seaweed extract [SW; 1% (v/v), 3 mL·L^-1^], humic acid [HA; 1% (v/v), 3 mL·L^-1^], and a combined seaweed extract plus humic acid treatment [SW+HA; 1.5 + 1.5 mL·L^-1^]. The biostimulant doses were selected based on previous spinach studies conducted under greenhouse conditions, in which seaweed extract was applied at a comparable concentration as a foliar treatment (Rouphael et al., 2018) and humic acid was applied at an equivalent dose through the soil (Naseri et al., 2021). The seaweed extract product was Kelpak^®^ (Kelp Products International Pty Ltd., Cape Town, South Africa), derived from fresh *Ecklonia maxima* and containing 1.0% soluble potash (K_2_O). The humic acid product was Nutra Need™ enzyme biostimulant (Tainio Biologicals, Inc., Spokane, WA, USA), containing 1% humic acid, 10% liquid extract of non-living soil bacteria, yeast, and fungal cultures, and 89% distilled water. All treatment solutions were prepared using reverse osmosis (RO) water (EC = 0.005 dS·m^-1^; pH = 6.05). The EC and pH (mean ± SE, n = 8) of the prepared solutions were 0.12 ± 0.02 dS·m^-1^ and 6.27 ± 0.04 for SW, 0.10 ± 0.03 dS·m^-1^ and 6.29 ± 0.08 for HA, and 0.14 ± 0.03 dS·m^-1^ and 6.33 ± 0.03 for SW+HA, respectively.

**Figure 1:**
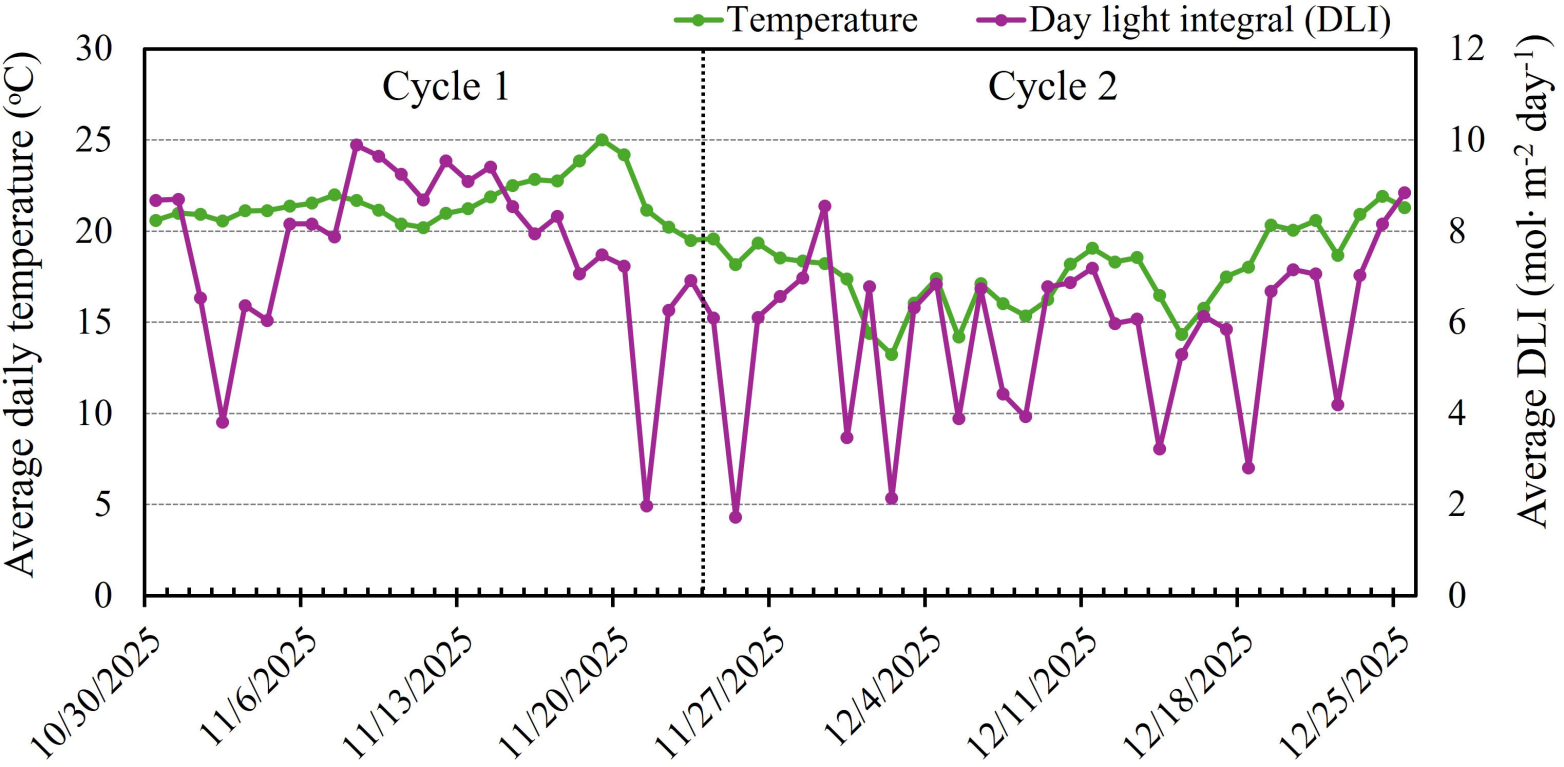
Daily average air temperature (green line, primary Y-axis) and daily light integral (DLI; purple line, secondary Y-axis) recorded in the greenhouse during the two experimental cycles. The vertical dashed line separates Cycle 1 and Cycle 2.

Two adjacent greenhouse benches with similar micro-environmental conditions were used as blocks to account for potential spatial variation. Adjacent benches were selected to accommodate the complete treatment layout while minimizing differences in environmental conditions across the treatments. Each block contained the complete set of biostimulant and cultivar combinations (**Supplementary Figure 1**). The layout shown in **Supplementary Figure 1** represents the initial arrangement of treatments at the start of the experiment. Plants were randomly assigned within treatment trays, and trays were repositioned within each bench three times per week throughout the experiment to minimize positional effects associated with light interception, temperature, and airflow. Individual plants were considered experimental units. In the first experimental cycle, six plants were used per cultivar and biostimulant treatment combination within each block, whereas four plants per combination were used in the second cycle because of lower germination.Biostimulant applications were conducted four times during each growth cycle at weekly intervals. For each treatment, plants were placed on trays (53.49 × 27.46 × 6.35 cm) and subirrigated by flooding with 900 mL of the respective treatment solution. After 3 to 4 h, once the substrate was fully saturated, excess solution was removed. Control plants were treated similarly using tap water. Between treatment applications, when the substrate began to dry, plants were subirrigated with the nutrient solution previously described for the propagation period. The nutrient solution was applied uniformly to all cultivars and biostimulant treatments at the same concentration and according to the same irrigation schedule; therefore, nutrient availability was consistent across all experimental units throughout the study. At this stage, the EC was slightly increased compared with the propagation phase, and across both experimental cycles the nutrient solution EC and pH averaged 1.43 ± 0.06 dS·m^-1^ and 6.3 ± 0.09, respectively (mean ± SE, n = 14). Greenhouse temperature and light conditions were recorded using a Campbell Scientific CR1000 datalogger. **Figure 1** illustrates the environmental conditions during the two experimental cycles. The average air temperature was 19.4 ± 2.6 °C, and the average daily light integral (DLI), calculated from daily mean light intensity using DLI = PPFD × 0.0864, was 19.4 ± 8.45 mol·m^-2^·d^-1^ (mean ± SE, n = 56). Plants were harvested on 26 and 31 days after treatment initiation in Cycle 1 (October 30 to November 24, 2026) and Cycle 2 (November 25 to December 25, 2026), respectively. Harvest in Cycle 2 was delayed because the average daily temperature and DLI were lower (average air temperature = 17.8 ± 2.2 °C, average DLI = 14.0 ± 5.9 mol·m^-2^·d^-1^, n = 31) than in Cycle 1 (average air temperature = 25.8 ± 6.2 °C, average DLI = 21.4 ± 1.5 mol·m^-2^·d^-1^, n = 26).

### 2.3 Data collection

#### 2.3.1 Growth and morphology

The following growth and morphological parameters were measured in both cycles at the time of harvest: shoot and root fresh weight (FW) and dry weight (DW), canopy diameter (CD), total leaf area (LA), third leaf length (LL), third leaf width (LW), and leaf number (LN). Plants were cut at the substrate level, and shoot FW was measured immediately using a digital balance. The intact root with substrate inside the pot was then stored at 4 °C in the dark for two days. Roots were subsequently washed with tap water, blotted dry, and weighed to determine root FW. Shoot samples were then dried in an oven at 80 °C for 48 h (Thermo Fisher Scientific, Waltham, MA, USA) and weighed again using the same balance to determine DW, CD, LL, and LW were measured using a ruler. Leaves were separated from the basal stem, and LA was measured using an LI-3100C Leaf Area Meter (LI-COR, Lincoln, NE, USA).

#### 2.3.2 Phytochemicals and antioxidant activity

Immediately after harvest, mid-sized leaves from each sample were selected, ground in liquid nitrogen with a mortar and pestle, and stored at -80 °C until analysis of phytochemicals and antioxidant activity. The resulting composite samples were used as biological replicates for subsequent analyses. Total chlorophyll (Chl a + Chl b), carotenoids, and anthocyanins were determined following Wellburn (1994) with slight modifications. Leaf tissue of 50 mg was extracted with 1.5 mL high-grade methanol in 2 mL amber tubes and incubated in darkness at 4 °C for 18 h. Samples were vortexed under low light and centrifuged (Eppendorf 5417R, Hamburg, Germany) at 10,000 rpm for 5 min at 10 °C. The supernatant (1 mL) was transferred to a 1-cm cuvette, and absorbance was measured at 665.2, 652.4, and 470 nm using a spectrophotometer (Genesys 10S UV-Vis, Thermo Scientific, Madison, WI, USA). Pigment concentrations were calculated using the equations of Wellburn (1994): Chl a (µg mL^-1^) = 16.72 × A665.2 - 9.16 × A652.4, Chl b (µg mL^-1^) = 34.09 × A652.4 - 15.28 × A665.2, Car (µg mL^-1^) = [1000 × A470 - 1.63 × Chl a - 104.96 × Chl b]/221; where A665.2, A652.4, and A470 represent the absorbance values measured at 665.2 nm, 652.4 nm, and 470 nm, respectively.

Anthocyanin, total phenolic content (TPC), total flavonoid content (TFC), and antioxidant activity determined by 2,2 diphenyl-1-picrylhydrazyl (DPPH) radical scavenging or inhibition activity were measured using the same extraction procedure. Approximately 200 mg of ground leaf tissue was extracted with 1.5 mL of 1% acidified methanol in 2 mL microtubes and incubated in darkness at 4 °C for 16 h. The extracts were centrifuged (Eppendorf 5417R microcentrifuge, Hamburg, Germany) at 10,000 rpm for 15 min, and the supernatant was used for subsequent analyses. Anthocyanin content was quantified according to Dou et al. (2019) by measuring absorbance at 520 nm using 1% acidified methanol as the blank and expressed as mg cyanidin-3-glucoside equivalents (CE) g^-1^ fresh weight using the formula: Anthocyanin (mg CE g^-1^ FW) = (V × M × A)/(ϵ × m), where V is extraction volume (mL), M is the molecular weight of cyanidin-3-glucoside (449.2 g mol^-1^), A is absorbance at 520 nm, ϵ is the molar extinction coefficient (29,600), and m is sample weight. Total phenolic and flavonoid contents were determined following Chew et al. (2011) and Tan et al. (2011), respectively. For TPC, 20 µL of extract was reacted with Folin-Ciocalteu reagent and sodium carbonate (Na_2_CO_3_), incubated for 2 h at room temperature approximately 25 °C, and absorbance was measured at 725 nm using a ELx800 microplate reader (BioTek, Winooski, VT, USA). For TFC, 20 µL of extract was reacted sequentially with sodium nitrite (NaNO_2_), aluminum chloride hexahydrate (AlCl_3_·6H_2_O), and sodium hydroxide (NaOH), and absorbance was measured at 520 nm. Blanks and standard curves were included in each assay. Results were expressed as mg gallic acid equivalent (GAE) g^-1^ fresh weight for TPC and mg catechin equivalent (CE) g^-1^ fresh weight for TFC.

DPPH radical scavenging activity was determined following Brand-Williams et al. (1995). A 0.1 mM DPPH solution was prepared in methanol, and 1 mL of the solution was mixed with 100 µL of plant extract. The reaction mixture was incubated in dark condition at 25 °C for 30 min, and absorbance was measured at 520 nm. Methanol served as the blank, and DPPH solution without extract served as the control. Percentage inhibition was calculated as: DPPH inhibition (%) = [(A_control_ - A_sample_)/A_control_] × 100; where A_control_ represents the absorbance of the control solution and A_sample_ represents the absorbance of the sample extract.

#### 2.3.3 Tissue mineral analysis

Dried leaf tissue samples from cycle 1 were ground using a Magic Bullet blender (Capital Brands, Los Angeles, CA, USA) to pass through a 40-mesh screen, and the resulting dry powder was used for mineral analyses. Samples were submitted to the Texas A&M AgriLife Extension Service Soil, Water, and Forage Testing Laboratory, College Station, TX, USA (30.6227° N, 96.3597° W). Total nitrogen (N) was determined by a high-temperature combustion method using an automated elemental analyzer and reported on a dry weight basis (Nelson and Sommers, 1973; Sheldrick, 1986; Sweeney, 1989; McGeehan and Naylor, 1988). Mineral elements including phosphorus (P), potassium (K), calcium (Ca), magnesium (Mg), sodium (Na), zinc (Zn), iron (Fe), manganese (Mn), sulfur (S), and boron (B) were determined by inductively coupled plasma (ICP) spectrometry following nitric acid digestion, and concentrations were expressed on a dry weight basis (Isaac and Johnson, 1975; Havlin and Soltanpour, 1980).

### 2.4 Statistical analysis

A two-way analysis of variance (ANOVA) was performed to evaluate the effects of biostimulant treatment, cultivar, and their interaction. When significant effects were detected, means were separated using Tukey’s honestly significant difference (HSD) test at P ≤ 0.05. The proportion of variance explained by the fitted ANOVA models was assessed using the coefficient of determination (R2), which was calculated for each response variable and reported in the corresponding ANOVA tables. In the absence of significant interactions, data were pooled across factors. In addition, one-way ANOVA was conducted within each cultivar to further examine treatment effects. Experimental cycle was initially included in the statistical model as a factor. However, cycle effects and all interactions involving cycle were not significant for any growth or morphological parameters. In addition, greenhouse environmental conditions were comparable between cycles (**Figure 1**). Although the total number of plants per block differed between cycles, an equal number of plants was sampled from each cycle for statistical analysis. Therefore, data from both cycles were pooled for subsequent analyses. Prior to ANOVA, residuals were tested for normality and homogeneity of variance using the Shapiro-Wilk and Levene’s tests, respectively, and the results are provided in the **Supplementary Table S1**. Additional parameters were measured during the experiment; however, only variables that satisfied ANOVA assumptions and were relevant to the study objectives are presented. Statistical analyses were conducted in RStudio (version 2024.12.1; Posit Software, Boston, MA, USA) using R (version 4.4.2; R Foundation for Statistical Computing, Vienna, Austria). ANOVA was performed using the stats package, mean separation using agricolae, and graphical visualization using ggplot2. Data were standardized using Z-score transformation [Z = (x - μ)/σ], where x is the observed value, μ is the mean, and σ is the standard deviation. Standardized data were used for heatmap generation with pheatmap and principal component analysis (PCA) with prcomp and visualized using factoextra. Positive and negative Z-scores indicate values above and below the dataset mean, respectively.

## 3 Results

### 3.1 Growth and morphological traits

Significant two-way interactive effects between biostimulant (BS) and cultivar (CV) were noticed for shoot FW, shoot DW, root FW, root DW, CD, and LA, whereas no interaction was observed for LL, LW, and LN (**Table 1**). Biostimulant treatments significantly influenced all growth and morphological traits. However, cultivar effect was not significant except for canopy diameter. The ANOVA models explained 33-55% of the observed variation across growth and morphological traits.

**Table 1:** Summary of analysis of variance (ANOVA) showing the effects of four biostimulant treatments [control (no biostimulant), seaweed extract (1% v/v applied at 3 mL·L^-1^), humic acid (1% v/v applied at 3 mL·L^-1^), and combined seaweed extract and humic acid (each applied at 1.5 mL·L^-1^)] and three spinach cultivars (‘Lakeside’, ‘Mandolin’, and ‘SV2157’) on growth and morphological traits, including shoot fresh weight (FW), shoot dry weight (DW), root FW, root DW, canopy diameter, total leaf area, leaf number, leaf length, and leaf width.

Factor	Shoot FW	Shoot DW	Root FW	Root DW	Canopy diameter	Leaf area	Leaf length	Leaf width	Leaf number
Biostimulant (BS)	***	***	***	***	***	***	***	***	***
Cultivar (CV)	***	***	***	***	NS	***	***	***	***
BS×CV	*	*	**	***	*	NS	NS	NS	NS
Variance explained (R^2^)	0.47	0.51	0.51	0.50	0.55	0.45	0.48	0.33	0.50

NS denotes not significant; *, **, and *** indicate significance at *p* levels of ≤0.05, 0.01, and 0.001, respectively. Variance explained (R^2^) represents the proportion of total variation accounted for by the full ANOVA model (biostimulant treatment, cultivar, and their interaction).

**Figure 2:**
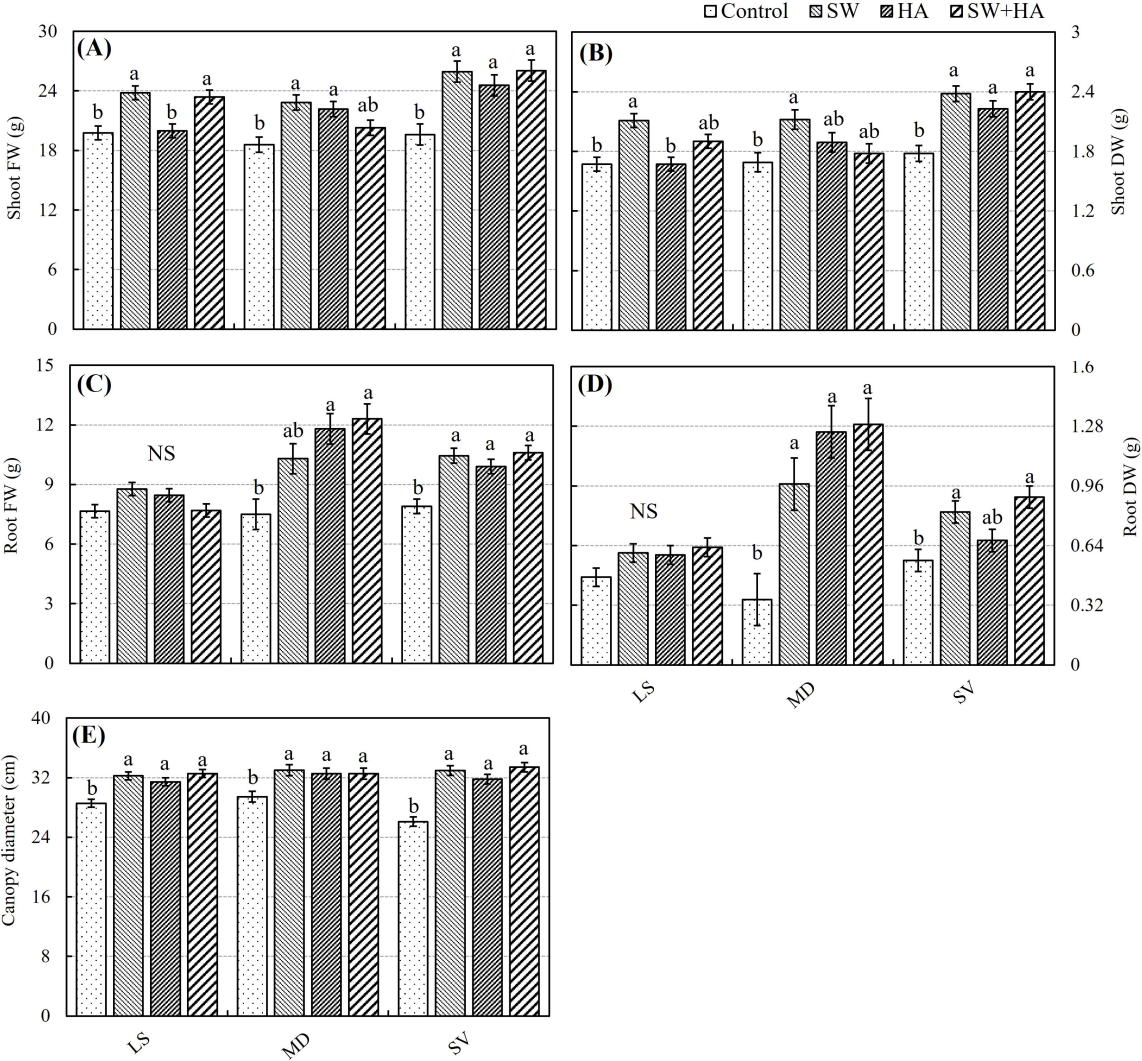
Shoot fresh weight (FW) **(A)**, shoot dry weight (DW) **(B)**, root FW **(C)**, root DW **(D)**, and canopy diameter **(E)** of three spinach cultivars (Lakeside: LS, Mandolin: MD, and SV2157: SV) in response to root-zone biostimulant treatments: Control (no biostimulant), seaweed extract (SW), humic acid (HA), and seaweed extract + humic acid (SW+HA) under greenhouse conditions. Bars represent means ± SE (n = 8). Different letters indicate significant differences among biostimulant treatments within a cultivar according to Tukey’s HSD test (P ≤ 0.05). NS indicates no significant differences.

In our study, growth responses to biostimulant treatments varied among the cultivars. In ‘Lakeside’, shoot FW increased by approximately 20% under SW and SW+HA compared to control and HA (**Figure 2A**). In ‘Mandolin’, SW and HA increased shoot FW by around 22%, while in ‘SV2157’ all treatments increased shoot FW by approximately 30% relative to control. Shoot DW increased in ‘Lakeside’ and ‘Mandolin’ only in response to SW, by 26% and 25%, respectively, in contrast to control (**Figure 2B**). Conversely, all biostimulant treatments increased shoot DW in ‘SV2157’, averaging 34% compared to control. Root FW and DW were unaffected by biostimulants in ‘Lakeside’ (**Figures 2C, D**). However, in ‘Mandolin’, HA and SW+HA increased root FW by an average of 37%, and all treatments significantly increased root DW, ranging from 177% to 268% compared to control. In ‘SV2157’, root FW increased by an average of 32% across treatments, while root DW increased by approximately 47% under SW and SW+HA relative to control. Canopy diameter (**Figure 2E**) increased significantly under all biostimulant treatments across the cultivars compared to the control.

**Figure 3:**
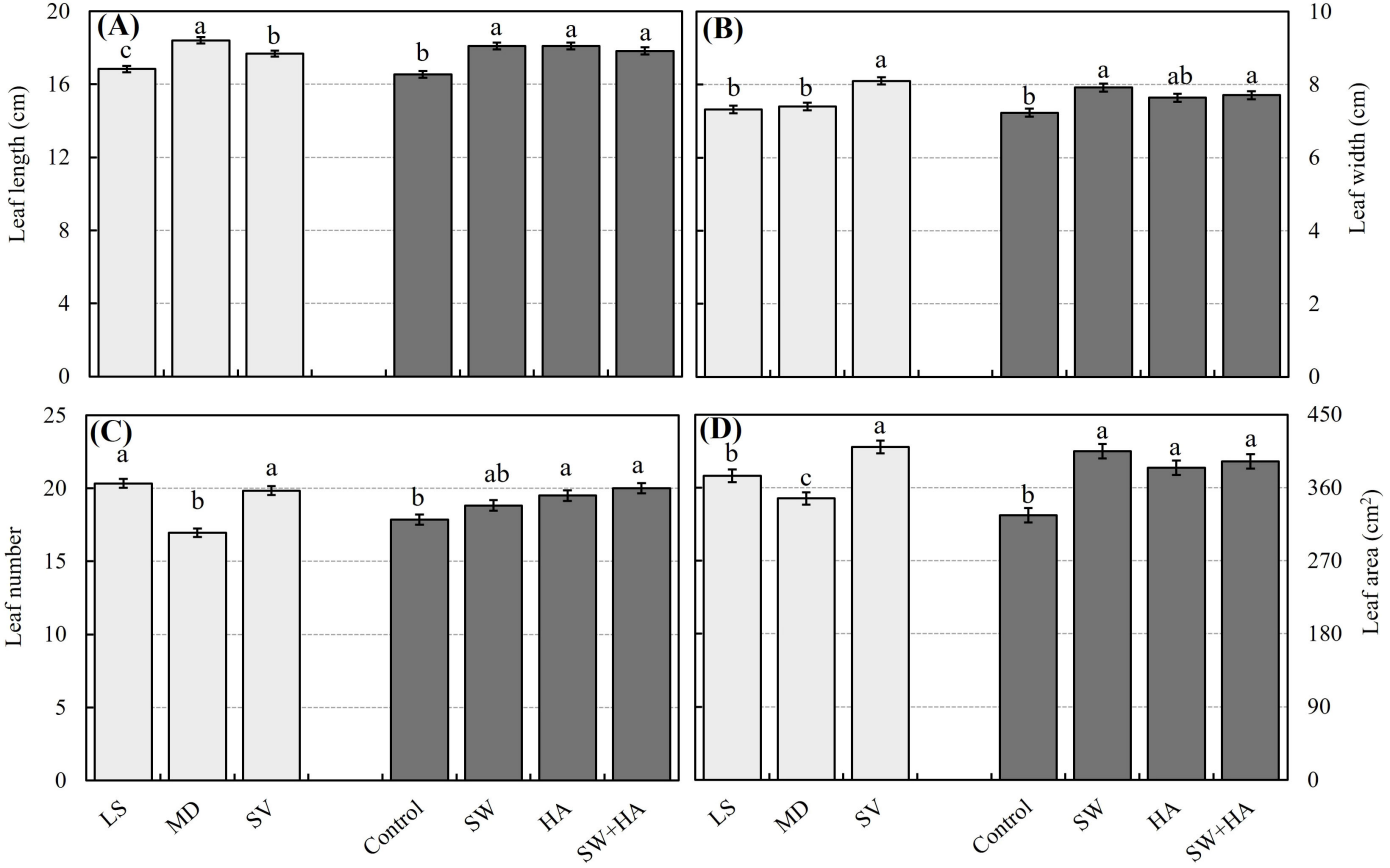
Individual effects on leaf characteristics of three spinach cultivars (Lakeside: LS, Mandolin: MD, and SV2157: SV) and biostimulant treatments [Control (no biostimulant), seaweed extract (SW), humic acid (HA), and seaweed extract + humic acid (SW+HA)] under greenhouse conditions, including leaf length **(A)**, leaf width **(B)**, leaf number **(C)**, and leaf area **(D)**. Bars represent means ± SE (n = 8). Different letters indicate significant differences among cultivars or biostimulant treatments according to Tukey’s HSD test (P ≤ 0.05).

Leaf morphological traits were primarily cultivar dependent (**Figure 3**), with biostimulant treatments having selective and trait-specific effects. ‘Mandolin’ had the greatest leaf length (18.41 cm), and biostimulant treatments increased leaf length by approximately 12% compared with the control (**Figure 3A**). ‘SV2157’ exhibited the greatest leaf width (2.2 cm) compared with the other cultivars, with increases observed only in the SW and SW+HA treatments relative to the control (**Figure 3B**). ‘Lakeside’ and ‘SV2157’ had the highest leaf numbers (**Figure 3C**), which increased only under HA and SW+HA compared with the control. ‘SV2157’ exhibited the greatest leaf area (410.02 cm^2^) among the cultivars (**Figure 3D**), and all biostimulant treatments increased leaf area by approximately 20% relative to the control.

### 3.2 Phytochemical attributes and DPPH radical scavenging activity

For biochemical parameters, significant interactive effects between biostimulant and cultivar (BS × CV) were observed only for total anthocyanins and total phenolics (**Table 2**). Biostimulant treatments also significantly affected total chlorophylls, total carotenoids, and total anthocyanins, whereas cultivar effects were significant for all biochemical traits except DPPH inhibition activity. The ANOVA models explained a large proportion of the variation in total chlorophylls (R2 = 0.83), total carotenoids (R2 = 0.75), total anthocyanins (R2 = 0.95), and total phenolics (R2 = 0.69), whereas lower explanatory power was observed for total flavonoids (R2 = 0.43) and particularly DPPH inhibition activity (R2 = 0.06).

**Table 2:** Summary of analysis of variance (ANOVA) showing the effects of four biostimulant treatments [control (no biostimulant), seaweed extract (1% v/v applied at 3 mL·L^-1^), humic acid (1% v/v applied at 3 mL·L^-1^), and combined seaweed extract and humic acid (each applied at 1.5 mL·L^-1^)] and three spinach cultivars (‘Lakeside’, ‘Mandolin’, and ‘SV2157’) on biochemical parameters, including total chlorophylls, total carotenoids, total anthocyanins, total flavonoids, total phenolics, and DPPH inhibition.

Factor	Total chlorophylls	Total carotenoids	Total anthocyanins	Total flavonoids	Total phenolics	DPPH inhibition
Biostimulant (BS)	*	***	***	NS	NS	NS
Cultivar (CV)	***	***	***	***	***	NS
BS×CV	NS	NS	***	NS	***	NS
Variance explained (R^2^)	0.83	0.75	0.95	0.43	0.69	0.06

NS denotes not significant; *, **, and *** indicates significance at P levels of ≤0.05, 0.01, and 0.001, respectively. Variance explained (R²) represents the proportion of total variation accounted for by the full ANOVA model (biostimulant treatment, cultivar, and their interaction).

**Figure 4:**
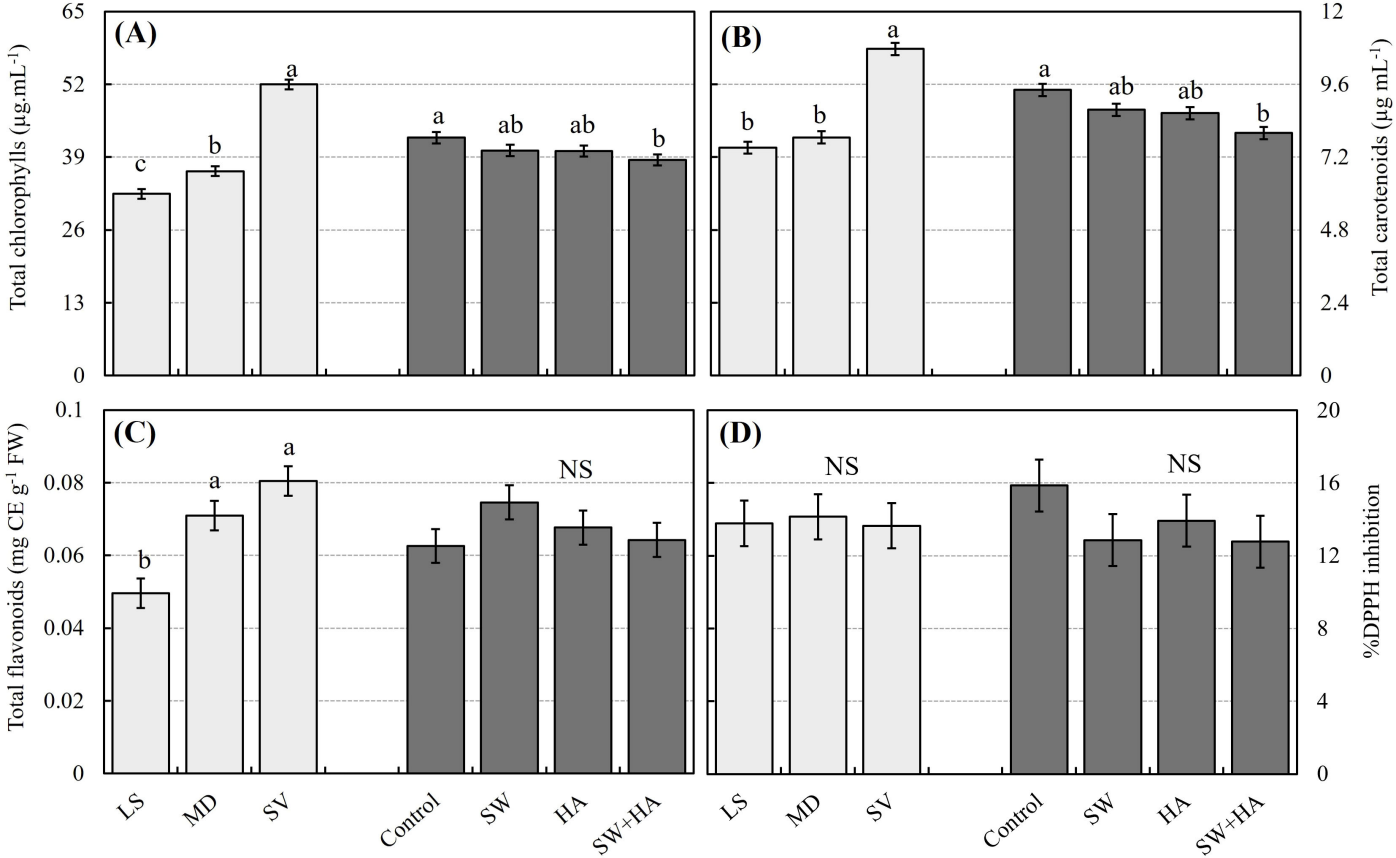
Individual effects of three cultivars (Lakeside: LS, Mandolin: MD, and SV2157: SV) and biostimulant treatments: Control (no biostimulant), seaweed extract (SW), humic acid (HA), and seaweed extract + humic acid (SW+HA)] on total chlorophylls **(A)**, total carotenoids **(B)**, total flavonoids **(C)**, and DPPH inhibition activity **(D)** under greenhouse conditions. Bars represent means ± SE (n = 8). Different letters indicate significant differences among cultivars or biostimulant treatments according to Tukey’s HSD test (P ≤ 0.05). NS indicates no significant differences.

Total chlorophyll and carotenoid contents were cultivar specific (**Figures 4A, B**). Among the cultivars, ‘SV2157’ exhibited the highest total chlorophyll (52.01 µg mL^-1^) and carotenoid concentration (10.76 µg mL^-1^). Biostimulant effects showed that, under the SW+HA treatment, both pigments were significantly reduced by approximately 9% for chlorophylls and 15% for carotenoids compared with the control.

**Figure 5:**
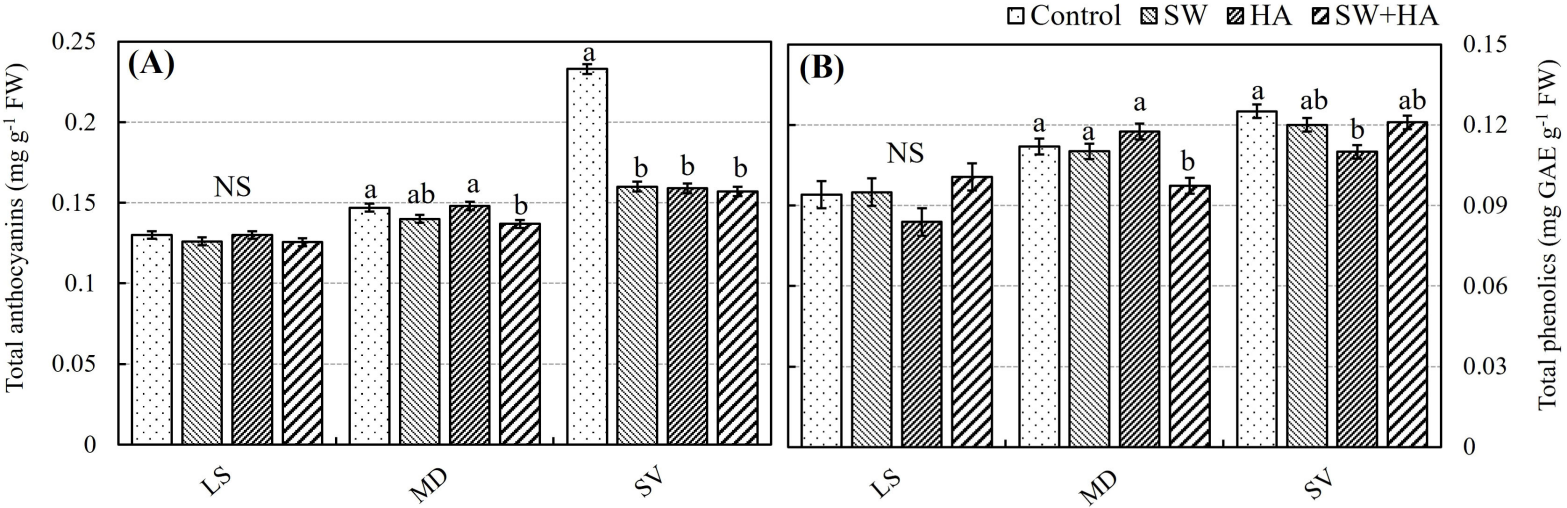
Total anthocyanins **(A)** and total phenolics **(B)** of three spinach cultivars (Lakeside: LS, Mandolin: MD, and SV2157: SV) in response to biostimulant treatments: Control (no biostimulant), seaweed extract (SW), humic acid (HA), and seaweed extract + humic acid (SW+HA) under greenhouse conditions. Bars represent means ± SE (n = 8). Different letters indicate significant differences among biostimulant treatments within a cultivar according to Tukey’s HSD test (P ≤ 0.05). NS indicates no significant differences.

On the other hand, biostimulant treatments had limited effects on TFC, DPPH inhibition activity, anthocyanin, and TPC regardless of cultivar (**Figures 4C, D**, **5**). Individual biostimulant applications did not significantly influence TFC, although ‘SV2157’ and ‘Mandolin’ exhibited higher TFC than ‘Lakeside’. Similarly, no significant differences in antioxidant activity were observed among cultivars or biostimulant treatments. Anthocyanin content was unaffected by biostimulant treatments in ‘Lakeside’. In contrast, the SW+HA treatment resulted in the lowest anthocyanin content in ‘Mandolin’, while in ‘SV2157’, anthocyanin content decreased by approximately 31% on average across all biostimulant treatments relative to the control. A similar trend was observed for TPC, where the SW+HA treatment resulted in lower TPC in ‘Mandolin’ compared with the other biostimulant treatments, whereas in ‘SV2157’, HA resulted in lower TPC than the control.

### 3.3 Minerals accumulation

Mineral nutrient accumulation exhibited significant BS × CV interactions for N, P, Ca, Mg, Na, Zn, Fe, and B, while K, Mn, and S had no interactive effects (**Table 3**). Biostimulant treatments significantly influenced all mineral nutrients except Fe, and cultivar effects were significant for all minerals except K. The fitted ANOVA models accounted for 58-98% of the variation in mineral concentrations. Variance explained was highest for Zn, S, N, Na, and Fe (R2 ≥ 0.92), consistent with the strong effects of biostimulant treatment, cultivar, and their interactions on nutrient accumulation, whereas K and Mn showed relatively lower model explanatory power (R² = 0.58 and 0.64, respectively).

**Table 3:** Summary of analysis of variance (ANOVA) showing the effects of four biostimulant treatments [control (no biostimulant), seaweed extract (1% v/v applied at 3 mL·L^-1^), humic acid (1% v/v applied at 3 mL·L^-1^), and combined seaweed extract and humic acid (each applied at 1.5 mL·L^-1^)] and three spinach cultivars (‘Lakeside’, ‘Mandolin’, and ‘SV2157’) on mineral nutrient concentrations, including nitrogen (N), phosphorus (P), potassium (K), calcium (Ca), magnesium (Mg), sodium (Na), zinc (Zn), iron (Fe), manganese (Mn), sulfur (S), and boron (B).

Factor	N	P	K	Ca	Mg	Na	Zn	Fe	Mn	S	B
Biostimulant (BS)	***	*	**	*	**	***	***	NS	**	***	***
Cultivar (CV)	***	***	NS	***	***	***	***	***	***	***	**
BS×CV	***	***	NS	***	***	***	***	***	NS	NS	**
Variance explained (R^2^)	0.96	0.89	0.58	0.75	0.85	0.96	0.98	0.92	0.64	0.97	0.90

NS denotes not significant; *, **, and *** indicates significance at P levels of ≤0.05, 0.01, and 0.001, respectively. Variance explained (R²) represents the proportion of total variation accounted for by the full ANOVA model (biostimulant treatment, cultivar, and their interaction).

**Figure 6:**
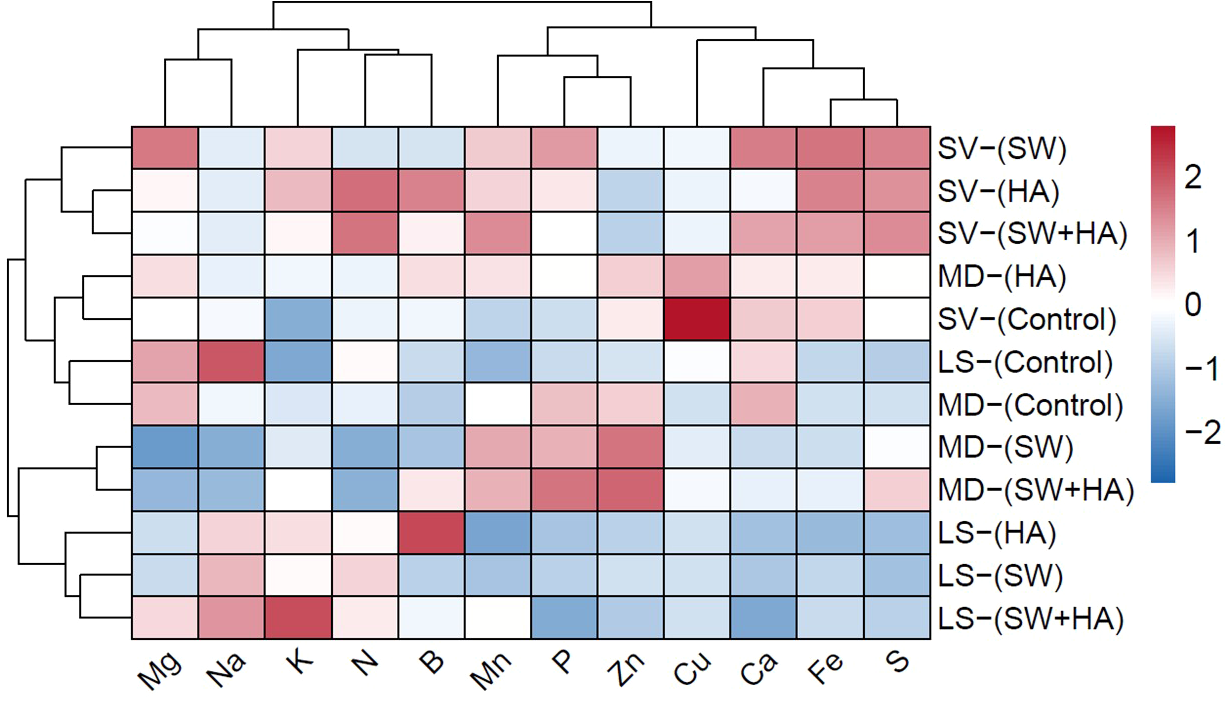
Z-score standardized heatmap of accumulation of minerals (N, P, K, Ca, Mg, S, Na, Zn, Fe, Cu, Mn, and B) of three spinach cultivars (‘Lakeside’: LS, ‘Mandolin’: MD, and ‘SV2157’: SV) in response to root-zone biostimulant treatments [control (no biostimulant), seaweed extract (1% v/v applied at 3 mL·L^-1^), humic acid (1% v/v applied at 3 mL·L^-1^), and combined seaweed extract and humic acid (each applied at 1.5 mL·L^-1^)] under greenhouse conditions. The color scale represents Z-score standardized values, where values were normalized relative to the mean concentration of each mineral across all cultivars and biostimulant treatments combinations. Red indicates values higher than the overall mean, blue indicates values lower than the mean, and white represents values close to the mean.

The Z-score standardized heatmap analysis revealed clear cultivar and treatment dependent mineral uptake patterns (**Figure 6**, **Supplementary Figure 2**). In ‘SV2157’, all biostimulant treatments resulted in predominantly positive Z-scores for multiple minerals compared with the control. SW treatment resulted in strong enrichment of Ca (Z = 1.56), Mg (Z = 1.61), Fe (Z = 1.65), Mn (Z = 0.65), and S (Z = 1.49). HA treatment increased N (Z = 1.73), K (Z = 0.84), Fe (Z = 1.48), S (Z = 1.32), and B (Z = 1.47), whereas the SW+HA treatment resulted in a more balanced enrichment across N (Z = 1.64), Ca (Z = 1.11), Fe (Z = 1.18), Mn (Z = 1.37), and S (Z = 1.37). In contrast, the control treatment showed negative or near zero Z-scores for most minerals except Cu (Z = 2.79).

In ‘Mandolin’, mineral responses were more selective. SW and SW+HA treatments increased Zn (Z = 1.68 to 1.82) and Mn (Z = 0.94 to1.07) but were associated with negative Z-scores for N (Z = -1.42 to -1.50) and Mg (Z = -1.33 to -1.84). In ‘Lakeside’, limited mineral plasticity across treatments was observed; although K increased under SW+HA (Z = 2.11) and B increased under HA (Z = 2.18), Ca, Fe, and Mn remained consistently reduced across treatments (Z ≤ -0.73).

**Figure 7:**
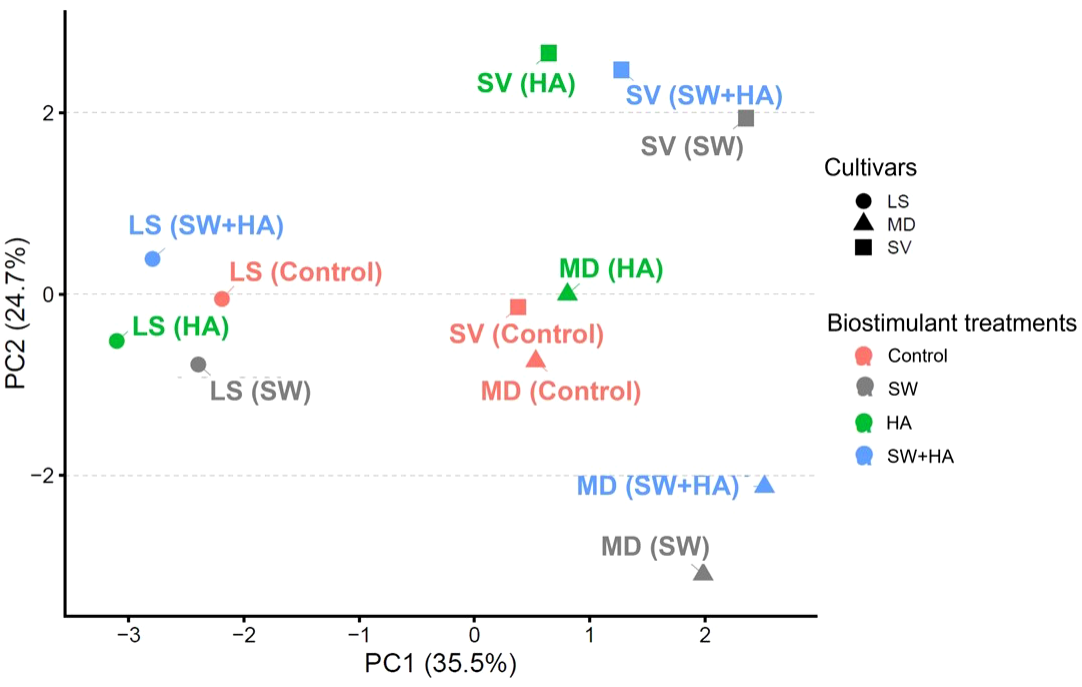
Principal component analysis (PCA) of mineral content (N, P, K, Ca, Mg, S, Na, Zn, Fe, Cu, Mn, and B) of three spinach cultivars (‘Lakeside’: LS, ‘Mandolin’: MD, and ‘SV2157’: SV) in response to root-zone biostimulant treatments [control (no biostimulant), seaweed extract (1% v/v applied at 3 mL·L^-1^), humic acid (1% v/v applied at 3 mL·L^-1^), and combined seaweed extract and humic acid (each applied at 1.5 mL·L^-1^)] under greenhouse conditions. PC1 and PC2 explain 35.5% and 24.7% of the total variance, respectively. Symbols represent cultivars, and colors indicate biostimulant treatments.

Principal component analysis supported the heatmap results by summarizing multivariate mineral variation among BS × CV combinations (**Figure 7**). The first two principal components explained 60.2% of the total variance, with PC1 accounting for 35.5% and PC2 accounting for 24.7%. Samples separated primarily by cultivar along PC1, with ‘Lakeside’ clustering on the negative side, ‘Mandolin’ on the positive side, and ‘SV2157’ occupying an intermediate to positive range. Within ‘SV2157’, SW, HA, and SW+HA treatments clustered closely and were clearly separated from the control, indicating a strong and consistent multivariate mineral response to biostimulant application. In contrast, treatments in ‘Lakeside’ remained closely grouped, while ‘Mandolin’ exhibited greater dispersion among treatments.

## 4 Discussion

### 4.1 Cultivar-specific mineral coordination determines shoot and root growth, while leaf traits had limited response to biostimulant applications

In our study, root-zone application of SW and HA increased shoot and root growth; however, the extent of these responses differed among cultivars. Significant interactive effects between biostimulant and cultivar for shoot FW, shoot DW, root FW, root DW, canopy diameter, and leaf area, indicating that treatment effects were genotype dependent rather than uniform across spinach cultivars.

In ‘SV2157’, all biostimulant treatments increased shoot DW, while SW and SW+HA also increased root DW, indicating coordinated responses between root development, mineral acquisition, and shoot growth. Similar growth promoting effects of seaweed extracts have been reported in greenhouse studies in tomato and spinach (Ali et al., 2016; Colla et al., 2017; Rouphael et al., 2018). These responses are often attributed to hormone-like bioactive compounds (e.g., auxins and cytokinins) present in seaweed extracts, which stimulate root development and enhance nutrient uptake (Khan et al., 2009; du Jardin, 2015). Likewise, root-zone application of humic acid has been shown to enhance root development, nutrient acquisition, and biomass production in greenhouse-grown onion and tomato (Naseri et al., 2021; Forotaghe et al., 2021; Alenazi and Khandaker, 2021). Previous studies have attributed these responses to the ability of humic substances to stimulate root H^+^-ATPase activity and root proliferation, thereby improving nutrient uptake and utilization efficiency (Canellas et al., 2015). Consistent with these mechanisms, HA treatment in ‘SV2157’ enhanced the accumulation of several essential minerals, particularly N, K, Fe, S, and B, which coincided with improved shoot biomass. Because the commercial humic acid-based biostimulant used in this study also contained extracts from non-living bacterial, fungal, and yeast cultures, these microbial-derived compounds may have provided additional bioactive molecules or signaling compounds; however, their specific contribution cannot be distinguished from the effects of humic substances alone. Similarly, SW treatment promoted greater accumulation of Ca, Mg, Fe, Mn, and S, further supporting improved nutrient acquisition associated with enhanced plant growth. Increased concentrations of Ca, S, and B further indicate enhanced structural integrity and metabolic capacity (Livorness and Smith, 2007; Kroh and Pilon, 2020; Hawkesford et al., 2023). Accordingly, the PCA showed a clear separation between biostimulant-treated plants and the control, indicating a consistent treatment induced shift in the mineral profile. Notably, although SW+HA promoted balanced multi-element enrichment in ‘SV2157’, its effects did not consistently exceed those of the individual treatments. This suggests that SW+HA had complementary yet partially overlapping mechanisms rather than strong synergistic enhancement. Because both seaweed extracts and humic substances can influence membrane transport processes and nutrient assimilation pathways (Khan et al., 2009; Canellas et al., 2015), their combined application may have stimulated similar nutrient uptake mechanisms. Once nutrient demand was sufficiently met, additional stimulation likely provided limited benefits for biomass accumulation, which may explain the absence of consistent additive effects under SW+HA.

Conversely, in both ‘Mandolin’ and ‘Lakeside’, increased root growth was not consistently coordinated with mineral accumulation and shoot biomass responses. In ‘Mandolin’, HA and SW+HA increased root DW, but shoot DW increased only under SW. Under HA and SW+HA, leaf N and Mg concentrations were lower, suggesting a dilution effect where increased root growth was not accompanied by sufficient nutrient accumulation in shoot tissues. Nitrogen deficiency reduces Rubisco and other photosynthetic proteins, and Mg deficiency limits chlorophyll formation and carbon fixation (Hermans et al., 2006; Cakmak and Kirkby, 2008; Kang et al., 2022). As a result, higher root biomass did not translate into proportional shoot DW because photosynthetic capacity was constrained. The contrasting responses between ‘Mandolin’ and ‘SV2157’ indicate that the effectiveness of the commercial HA-based biostimulant was dependent on the cultivar’s ability to coordinate root development with nutrient acquisition and subsequent shoot growth. Although microbial-derived compounds in the commercial formulation may have contributed to root-associated responses, their individual role remains uncertain and requires further investigation.

Moreover, in ‘Lakeside’, biostimulants had no significant effects on root growth, and only limited changes in mineral composition were observed compared with the control. Notably, leaf Ca, Fe, and Mn remained consistently low across treatments. Because these nutrients contribute to photosynthetic function and structural development, their limited accumulation may have restricted increases in shoot and root DW despite biostimulant application. PCA clustering also indicated low mineral uptake in this cultivar, suggesting a limited ability to adjust nutrient uptake in response to biostimulants. This limited responsiveness further demonstrates that successful biostimulant-induced growth depends on the cultivar’s capacity to translate changes in root activity into efficient nutrient acquisition and shoot development.

Nonetheless, leaf traits (leaf length, width, and number) were mostly determined by cultivar and showed no significant interaction with biostimulant treatments. This contrasts with Naseri et al. (2021), who reported increased leaf number following humic acid application in spinach, possibly reflecting differences in cultivar response or greenhouse conditions. In the present study, canopy diameter and leaf area increased with biomass, whereas leaf length, width, and number remained relatively unchanged. This suggests that root-zone biostimulants primarily enhance biomass accumulation without substantially altering genetically determined leaf architecture.

Root-zone biostimulant treatments were most effective when root development, mineral accumulation, and shoot biomass were closely coordinated, as observed in ‘SV2157’, and less effective when this coordination was incomplete, as in ‘Mandolin’, or limited, as in ‘Lakeside’. Therefore, the effectiveness of SW and HA depended more on the cultivar’s ability to adjust nutrient acquisition and allocation than on biostimulant application alone. Because biomass enhancement was closely associated with improved nutrient coordination, it was important to determine whether these responses in primary metabolism were accompanied by changes in secondary metabolism.

### 4.2 Growth enhancement occurred without activation of secondary metabolic pathways

Although SW and HA increased shoot biomass and mineral accumulation, their effects on secondary metabolites were limited. Significant biostimulant and cultivar interactive effects were observed only for anthocyanin and total phenolics, while total flavonoids and DPPH inhibition activity were unaffected. This indicates that root-zone biostimulant applications primarily enhanced primary metabolism rather than functioning as stress elicitors that broadly stimulate secondary metabolism under greenhouse conditions. Similar responses have been reported in lettuce and baby rocket, where biostimulants increased specific antioxidant-related compounds but did not consistently enhance overall antioxidant capacity, suggesting that antioxidant responses depend on crop species, nutrient availability, and growing conditions (Di Mola et al., 2019; Turan et al., 2026).

In ‘SV2157’, anthocyanin concentration decreased across biostimulant treatments despite substantial increases in shoot dry weight and mineral uptake. Anthocyanins commonly accumulate under environmental stress, such as high light intensity, nutrient imbalance, or oxidative pressure, where they function in photoprotection and reactive oxygen species scavenging (Chalker-Scott, 1999; Khan et al., 2025b). The reduction observed here likely reflected improved nutritional balance and reduced internal stress signaling. When nutrient availability is adequate and carbon assimilation is efficient, plants tend to allocate assimilates toward structural growth rather than defensive metabolite production, consistent with the growth differentiation balance hypothesis (Herms and Mattson, 1992). Likewise, TPC and antioxidant activity (DPPH) did not increase in response to biostimulant treatments. Secondary metabolite synthesis is often driven by stress-related signaling rather than enhanced nutrient availability alone (Zoratti et al., 2014). Although the commercial HA formulation contained microbial-derived extracts that may participate in defense-related signaling pathways, the inconsistent anthocyanin and phenolic responses among cultivars indicate that any contribution was cultivar dependent and could not be separated from the effects of humic substances alone. Because the experiment was conducted under controlled conditions without major abiotic stress, improved root function and nutrient acquisition likely supported primary metabolism without strongly activating oxidative defense pathways. This contrasts with Rouphael et al. (2018), who reported increased TPC and TFC following foliar application of seaweed extract in spinach. These differences suggest that biostimulant effects on secondary metabolism are influenced by application methods, growing conditions, stress intensity, and cultivar response.

Slight reductions in chlorophyll and carotenoid concentrations observed particularly under SW+HA, likely reflected a dilution effect associated with rapid biomass accumulation rather than inhibition of pigment synthesis. When leaf expansion and structural biomass increase disproportionately relative to pigment biosynthesis, pigment concentration per unit tissue can decline even though overall photosynthetic capacity per plant is maintained (Taiz et al., 2015). This pattern is consistent with enhanced growth supported by balanced mineral supply rather than pigment limitation.

In this study, root-zone application of SW and HA enhanced biomass primarily in ‘SV2157’ through improved nutrient accumulation and primary metabolic efficiency, while secondary metabolic pathways remained largely unaffected. Growth stimulation therefore appeared to occur through strengthened photosynthetic and nutritional processes rather than through activation of stress-induced antioxidant defenses. These results further support that cultivar-specific mineral uptake responses are a primary determinant of root-zone biostimulant effectiveness in spinach.

## 5 Conclusion

Our study demonstrated that root-zone application of commercial seaweed extract and a humic acid-based biostimulant containing non-living microbial-derived extracts enhanced spinach growth; however, the magnitude of these responses was strongly cultivar dependent. Growth promotion occurred when enhanced root development was accompanied by improved mineral accumulation and balanced nutrient acquisition supporting photosynthetic and metabolic processes, as observed in ‘SV2157’. In this cultivar, the combined application of seaweed extract and the humic acid-based biostimulant improved multi-element balance but did not consistently result in greater biomass than individual applications, suggesting complementary rather than synergistic effects. In contrast, increased root growth in ‘Mandolin’ and limited root responsiveness in ‘Lakeside’ were not consistently associated with enhanced nutrient accumulation or proportional increases in shoot dry weight. Leaf morphological traits remained largely cultivar dependent, while secondary metabolites and antioxidant activity showed limited responses, indicating that root-zone biostimulants primarily enhanced vegetative growth rather than stress-related metabolism under optimal greenhouse conditions. These findings demonstrate that the effectiveness of root-zone biostimulants in spinach depends on the capacity of individual cultivars to translate improved nutrient acquisition into biomass production. Therefore, cultivar selection should be considered alongside biostimulant application in commercial controlled environment production, as responsive cultivars such as ‘SV2157’ are more likely to achieve greater benefits from root-zone biostimulant treatments. Future studies should compare purified humic substances with commercial formulations containing microbial-derived extracts and evaluate different application rates and combinations of seaweed extract and humic acid-based biostimulants to clarify the contribution of individual components and optimize nutrient use efficiency and crop productivity.

## Data Availability

The raw data supporting the conclusions of this article will be made available by the authors, without undue reservation.

## Supplementary material

Supplementary Figure 1 Experimental layout of the randomized complete block design (RCBD) used in the greenhouse study. Two greenhouse benches served as blocks, each containing all four biostimulant treatments: Control (no biostimulant), seaweed extract (SW), humic acid (HA), and seaweed extract + humic acid (SW+HA). Each treatment included three spinach cultivars: Lakeside, Mandolin, and SV2157. The figure illustrates the initial arrangement of treatments at the start of the experiment.

Supplementary Figure 2 Z-score standardized values of mineral contents (N, P, K, Ca, Mg, S, Na, Zn, Fe, Cu, Mn, and B) of three spinach cultivars (‘Lakeside’: LS, ‘Mandolin’: MD, and ‘SV2157’: SV) in response to root-zone biostimulant treatments [control (no biostimulant), seaweed extract (1% v/v applied at 3 mL·L^-1^), humic acid (1% v/v applied at 3 mL·L^-1^), and combined seaweed extract and humic acid (each applied at 1.5 mL·L^-1^)] under greenhouse conditions.
