# Supplementary material for: Root-zone improvements in spinach through commercial biostimulants application vary among cultivars
Source: Front Plant Sci. 2026 Jul 13;17:1830913. doi: 10.3389/fpls.2026.1830913 (PMC13402458; doi:10.3389/fpls.2026.1830913)
Supplement: Supplementary Figure 1 — Experimental layout of the randomized complete block design (RCBD) used in the greenhouse study. Two greenhouse benches served as blocks, each containing all four biostimulant treatments: Control (no biostimulant), seaweed extract (SW), humic acid (HA), and seaweed extract + humic acid (SW+HA). Each treatment included three spinach cultivars: Lakeside, Mandolin, and SV2157. The figure illustrates the initial arrangement of treatments at the start of the experiment. [file DataSheet1.docx]

**Supplementary**
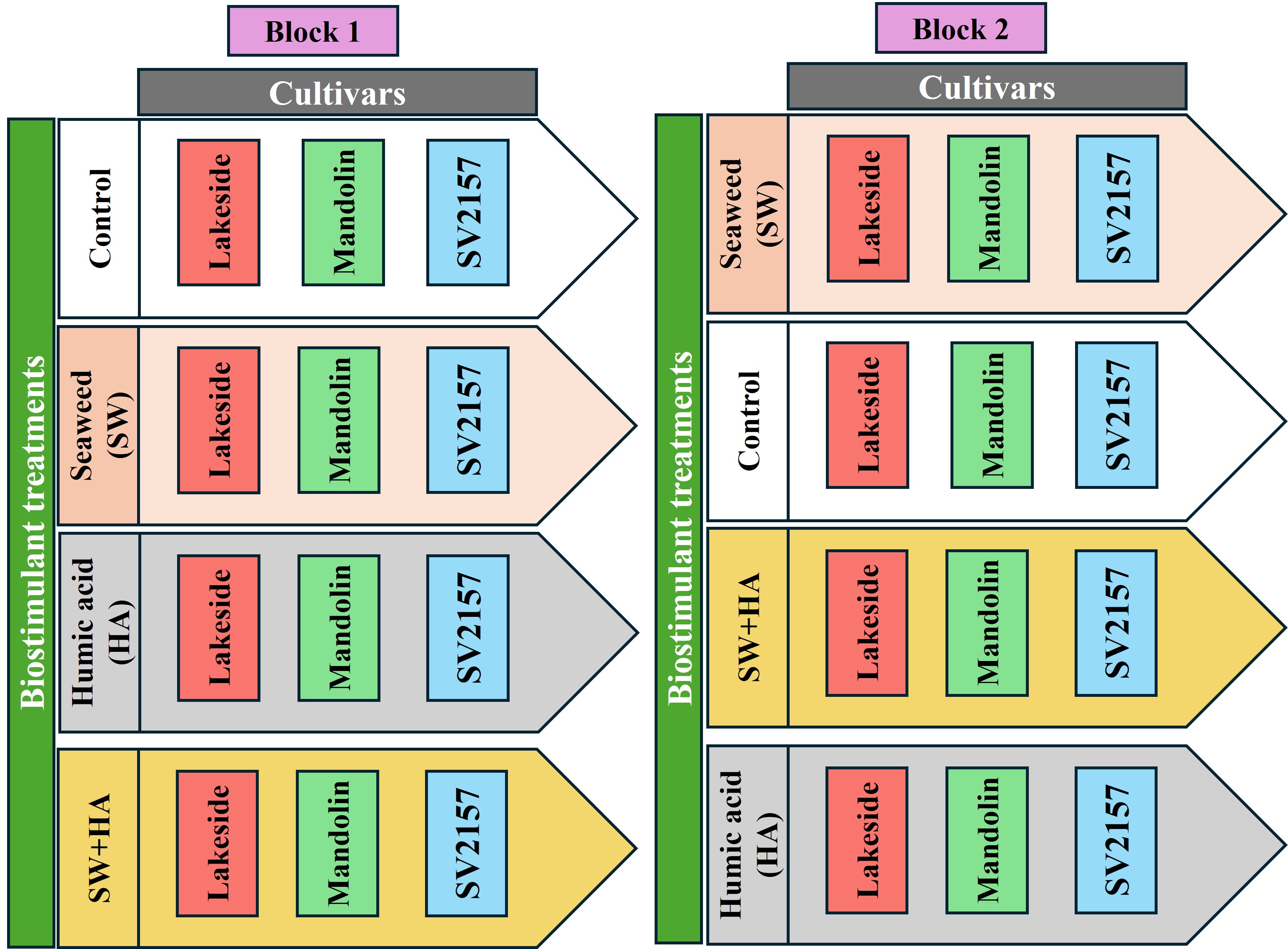
**figure S1.** Experimental layout of the randomized complete block design (RCBD) used in the greenhouse study. Two greenhouse benches served as blocks, each containing all four biostimulant treatments: Control (no biostimulant), seaweed extract (SW), humic acid (HA), and seaweed extract + humic acid (SW+HA). Each treatment included three spinach cultivars: Lakeside, Mandolin, and SV2157. The figure illustrates the initial arrangement of treatments at the start of the experiment.


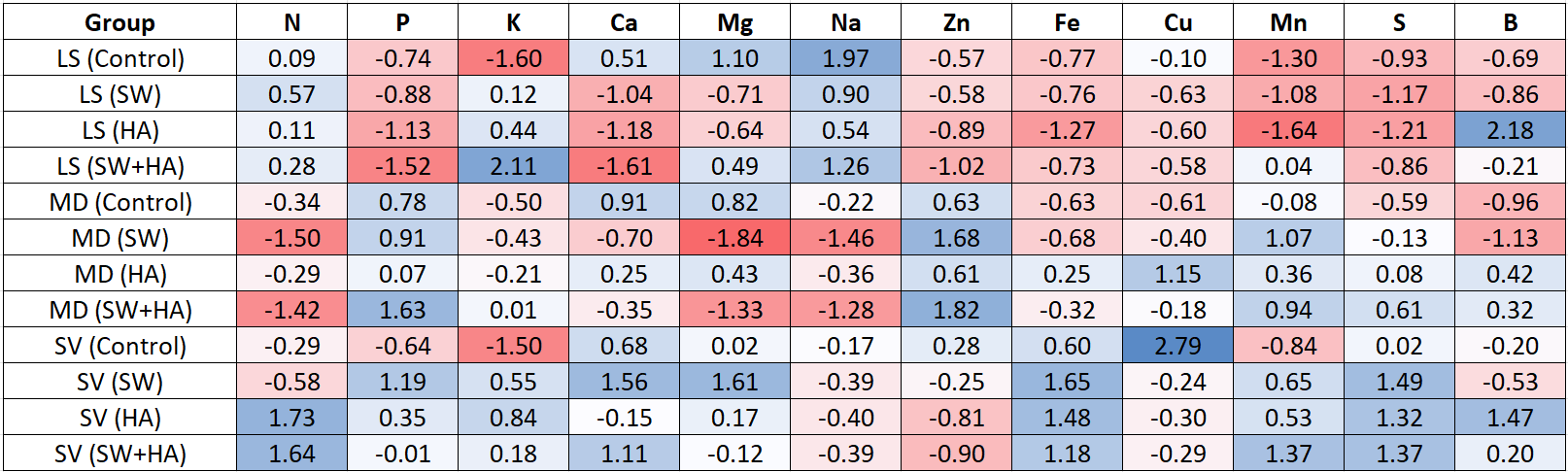


**Supplementary figure S2.** Z-score standardized values of mineral contents (N, P, K, Ca, Mg, S, Na, Zn, Fe, Cu, Mn, and B) of three spinach cultivars (‘Lakeside’: LS, ‘Mandolin’: MD, and ‘SV2157’: SV) in response to root-zone biostimulant treatments [control (no biostimulant), seaweed extract (1% v/v applied at 3 mL·L⁻¹), humic acid (1% v/v applied at 3 mL·L⁻¹), and combined seaweed extract and humic acid (each applied at 1.5 mL·L⁻¹)] under greenhouse conditions.

**Supplementary table S1.** Assessment of ANOVA assumptions for growth, morphological, pigment, phytochemical, and mineral nutrient variables measured in spinach. Normality and homogeneity of variance were evaluated using the Shapiro–Wilk and Levene's tests, respectively, based on residuals from the two-way ANOVA model. P-values˃ 0.05 indicate that the assumptions of normality and homogeneity of variance were satisfied.

| **Variable** | **Shapiro–Wilk P-value** | **Levene's P-value** |
| --- | --- | --- |
| Shoot FW | 0.0771 | 0.8075 |
| Shoot DW | 0.0759 | 0.7831 |
| Root FW | 0.3329 | 0.1105 |
| Root DW | 0.0782 | 0.1119 |
| Canopy diameter | 0.0875 | 0.1819 |
| Leaf area | 0.0806 | 0.8573 |
| Leaf length | 0.2504 | 0.4490 |
| Leaf width | 0.6396 | 0.1253 |
| Leaf number | 0.3305 | 0.2038 |
| Total chlorophylls (Chl) | 0.0881 | 0.5578 |
| Total carotenoids (Car) | 0.9111 | 0.0937 |
| Total anthocyanins (Ant) | 0.4129 | 0.4396 |
| Total phenolics (Phen) | 0.0999 | 0.7883 |
| Total flavonoids (Flv) | 0.8566 | 0.7119 |
| DPPH inhibition activity | 0.4024 | 0.0564 |
| N | 0.4261 | 0.8166 |
| P | 0.1198 | 0.4455 |
| K | 0.0412 | 0.6635 |
| Ca | 0.3451 | 0.1846 |
| Mg | 0.7982 | 0.8422 |
| Na | 0.9606 | 0.6205 |
| Zn | 0.0964 | 0.5055 |
| Fe | 0.2232 | 0.3193 |
| Mn | 0.0423 | 0.5537 |
| S | 0.8558 | 0.8413 |
| B | 0.1111 | 0.4612 |
